# Paraneoplastic Wong-Type Dermatomyositis Associated with Gynecological Malignancy

**DOI:** 10.3390/medicina58040484

**Published:** 2022-03-27

**Authors:** Roberto Russo, Giulia Gasparini, Emanuele Cozzani, Brunella Gravina, Aurora Parodi

**Affiliations:** 1 Department of Health Science (DISSAL), University of Genoa, 16132 Genoa, Italy; russoroberto@outlook.com (R.R.); gasparini.giulia@yahoo.it (G.G.); aurora.parodi@unige.it (A.P.); 2 Unit of Dermatology, San Martino Polyclinic Hospital, 16132 Genoa, Italy; 3 Department of Neurosciences, Rehabilitation, Ophthalmology, Genetics, Maternal and Child Health (DINOGMI), University of Genoa, 16132 Genoa, Italy; brunellagravina@virgilio.it

**Keywords:** dermatomyositis, Wong-type dermatomyositis, paraneoplastic skin diseases

## Abstract

Although dermatomyositis is known to be a possible paraneoplastic syndrome, often in the setting of gynecological cancers, Wong-type dermatomyositis—a rare variant of dermatomyositis—has not been clearly associated with internal malignancies to date. There is only one report from Japan of a woman who developed Wong-type dermatomyositis together with the recurrence of uterine cancer. We report the case of a Caucasian patient who presented with infrequent Wong-type dermatomyositis with positive anti-TIF1γ antibodies; screening for internal malignancies revealed fallopian tube carcinoma.

## 1. Introduction

Dermatomyositis (DM) is an inflammatory disease that affects skin and muscles [1]. Wong-type dermatomyositis (WTDM) is considered a rare subset of DM, presenting with erythematous hyperkeratotic follicular papules, mimicking pityriasis rubra pilaris [2]. Although some correlation between DM and cancer is widely accepted [1,3], the literature lacks reports of malignancy-associated WTDM.

## 2. Case Report

**Figure 1:**
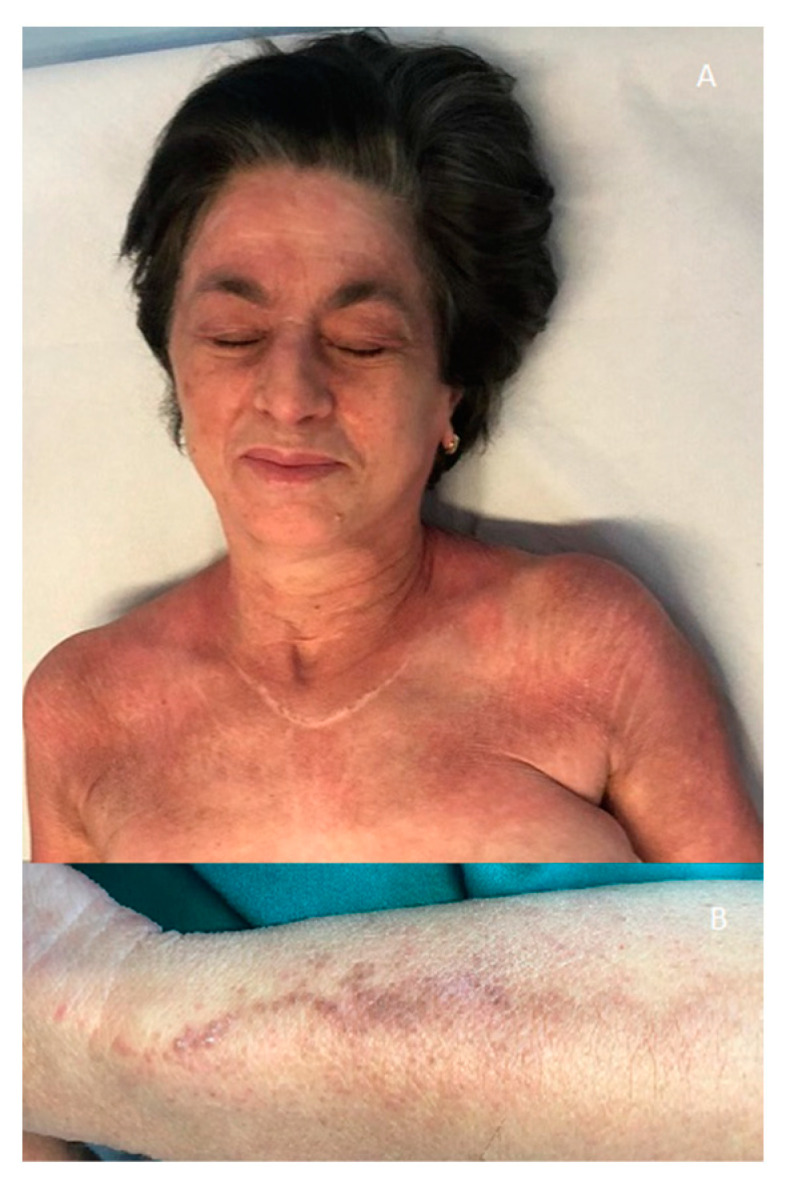
(**A**) Palpebral edema; heliotropic erythema of face, neck, chest, shoulder and arms. (**B**) Particular of hyperkeratotic, erythematous, follicular confluent papules arranged in a linear fashion on forearm.

**Figure 2:**
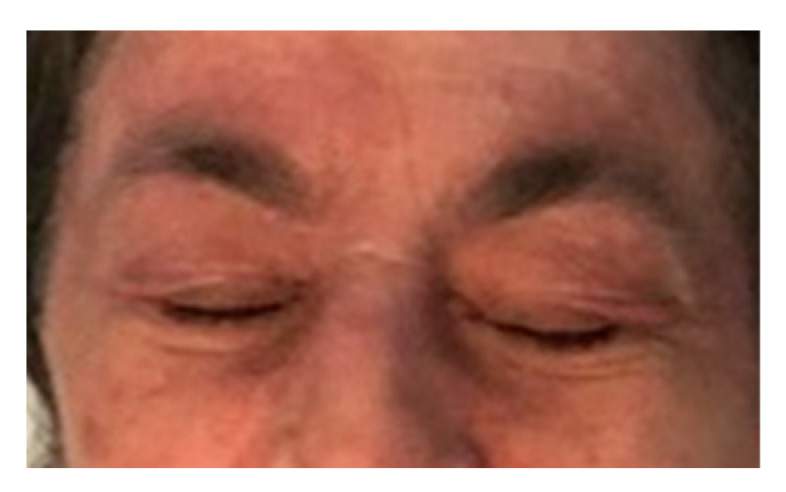
Close-up view of palpebral heliotropic erythema.

A 69-year-old Caucasian woman presented with a 2-month history of palpebral edema, heliotropic erythema of the face, neck, chest, shoulder and arms, Gottron papules and Gottron signs; hyperkeratotic, erythematous, follicular confluent papules arranged in a linear fashion were noted on the bony prominences of the chest, back and forearms (Figure 1 and Figure 2). The patient denied any muscular weakness. No anomalies were detected in laboratory exams including serum creatine kinase, lactic dehydrogenase, aldolase and transaminases. A myositis-specific antibodies test revealed positive anti-TIF1γ.

Clinical and laboratory findings allowed the diagnosis of amyopathic DM [4]. Hyperkeratotic, follicular, confluent, linearly arranged papules suggested WTDM [5]. A histological evaluation of a skin biopsy revealed follicular hyperkeratosis, keratotic plugs filling dilated follicular infundibula, vacuolar interface dermatitis and increased dermal mucin, confirming WTDM [2]. Systemic corticotherapy (prednisone 1 mg/kg) was administered with only mild response after 4 weeks. 

Since anti-TIF1γ positivity is often associated with underlying neoplasia [1], the patient was screened for malignancies. CT-scans of the abdomen revealed a solid lesion and a cystic lesion involving the right fallopian tube and ovarian. The patient underwent surgical excision of both fallopian tubes and ovaries, uterus and infracolic omentum, peritoneal washing and peritoneal biopsies. Histological examination revealed fallopian tube carcinoma, without macroscopic residual disease after surgery. Four weeks after surgery, dermatological evaluation revealed the remission of DM.

## 3. Discussion

WTDM is rare, as very few cases have been reported. It may occur in children and adults. Hyperkeratotic follicular papules and typical features of DM may overlap differently; when the former are prevalent, the diagnosis of WTDM may be delayed, as the clinical picture could be evocative of pityriasis rubra pilaris or other conditions with follicular hyperkeratosis [2]. Therefore, dermatologists should be very aware of this uncommon subset of DM, which in our opinion should be considered as a possible paraneoplastic dermatosis, similarly to typical DM. 

In fact, the prevalence of malignancy in patients with DM is assumed to be as high as 30% [1]. Gynecological cancers have been strongly associated with DM [3]. However, in the currently available literature, WTDM is not clearly associated with malignancies. In fact, Wong’s first report described 23 patients with DM, 52% of whom presented malignancy; however, only 11 of them were classified as WTDM, and the incidence of malignancy among them was not reported distinctly [6]. From then on, the only published report of malignancy-associated WTDM was a patient who developed WTDM simultaneously with the recurrence of uterine cancer; the cutaneous disease improved with corticotherapy, but the patient died a few months later because of metastatic disease [7]. Therefore, our report is the second one describing the overlap of WTDM with malignancy: interestingly, in both cases, there was an association with a gynecological cancer. Although a clear association between WTDM and malignancies have not been demonstrated in the literature, we believe that our report, together with the previous one [7], may allow us to propose WTDM as a possible paraneoplastic syndrome with a particular relationship with gynecological cancers; however, one should consider the fact that there are reports of WTDM with no associated malignancies [8]. Still, the well-known association between other subsets of DM and malignancies appears to endorse our hypothesis. The possible misdiagnosis and underreporting of this difficult-to-diagnose condition may explain the lack of similar reports so far. Dermatologists should be aware of the possibility of this correlation, avoid the pitfall of considering this clinical subset of DM as free from an association with cancer and screen their patients for malignancies as they do for other variants of DM.

## Data Availability

Not applicable.
